# 直链淀粉-三[(*S*)-1-苯乙基氨基甲酸酯]手性固定相拆分布地奈德对映体及其制剂含量的测定

**DOI:** 10.3724/SP.J.1123.2021.06048

**Published:** 2022-03-08

**Authors:** Yongpeng HUANG, Hui TANG, Xiangyan MENG, Bo CHEN, Hui ZHONG, Zhiyun ZOU

**Affiliations:** 国民核生化灾害防护国家重点实验室, 北京 102205; State Key Laboratory of NBC Protection for Civilian, Beijing 102205, China; 国民核生化灾害防护国家重点实验室, 北京 102205; State Key Laboratory of NBC Protection for Civilian, Beijing 102205, China; 国民核生化灾害防护国家重点实验室, 北京 102205; State Key Laboratory of NBC Protection for Civilian, Beijing 102205, China; 国民核生化灾害防护国家重点实验室, 北京 102205; State Key Laboratory of NBC Protection for Civilian, Beijing 102205, China; 国民核生化灾害防护国家重点实验室, 北京 102205; State Key Laboratory of NBC Protection for Civilian, Beijing 102205, China; 国民核生化灾害防护国家重点实验室, 北京 102205; State Key Laboratory of NBC Protection for Civilian, Beijing 102205, China

**Keywords:** 手性固定相, 高效液相色谱, 对映体, 布地奈德, chiral stationary phase, high performance liquid chromatography (HPLC), enantiomers, budesonide

## Abstract

22*R*-布地奈德的药物活性比22*S*-布地奈德的强2~3倍,开发布地奈德对映体拆分和定量分析方法,可为其药物研发及质量控制提供重要依据。目前,主要以反相C_18_固定相对布地奈德对映体进行拆分,而采用手性固定相对其进行拆分少有报道。通过考察固定相、流动相和柱温对布地奈德对映体拆分的影响,建立了基于直链淀粉-三[(*S*)-1-苯乙基氨基甲酸酯]手性固定相快速拆分和检测布地奈德对映体的高效液相色谱方法,其色谱条件如下:色谱柱为Chiralpak AS-RH色谱柱(150 mm×4.6 mm, 5.0 μm),流动相为乙腈-水(45:55, v/v),柱温40 ℃,流速1.0 mL/min,二极管阵列检测器(DAD),检测波长246 nm,进样量10 μL。在该色谱条件下,布地奈德的两个对映体得到较好拆分,22*R*-布地奈德和22*S*-布地奈德的保留时间分别6.40 min和7.77 min,分离度为4.64; 22*R*-布地奈德和22*S*-布地奈德分别在各自范围内线性关系良好,相关系数(*R*^2^)均为0.9999,检出限分别为0.05 μg/mL和0.07 μg/mL,定量限分别为0.16 μg/mL和0.20 μg/mL; 4个添加水平的样品加标回收率为102.63%~104.17%,相对标准偏差(RSD)为0.08%~0.57%(*n*=6)。将该方法应用于1批次4个吸入用布地奈德混悬液实际样品进行检测,22*R*-布地奈德和22*S*-布地奈德的含量分别为283.15~284.63 μg/mL和259.86~261.51 μg/mL。该方法操作简便,分析时间短,重复性好,准确度高,可用于布地奈德对映体的拆分及其制剂的质量控制。

## Abstract (en)

The drug budesonide exists as 22*R* and 22*S* enantiomers. However, the drug activity of 22*R*-budesonide is 2-3 times stronger than that of 22*S*-budesonide. The development of enantiomeric separation and quantitative analysis methods for budesonide can provide an important basis for its drug development and quality control. At present, the enantiomers of budesonide are separated on a reversed C_18_ solid phase column. However, chiral stationary phases are rarely reported for the separation of the enantiomers of budesonide. In this study, a high performance liquid chromatography (HPLC) method with a chiral stationary phase was developed for the rapid separation and determination of budesonide enantiomers. The effects of the type of chiral stationary phase, mobile phase additives, and column temperature on the resolution of the budesonide enantiomers were also investigated. The results showed that the chiral stationary phase amylose-tris-[(*S*)-1-phenylethyl carbamate] was more suitable for the separation of budesonide enantiomers. The mobile phase additives used in the experiment had no significant effect on the chromatographic parameters (peak height, peak width, and resolution) of the budesonide enantiomers. However, with an increase in the column temperature, the peak width of the budesonide enantiomers decreased, while the peak height and resolution increased. The optimized HPLC conditions were as follows: column, Chiralpak AS-RH (150 mm×4.6 mm, 5.0 μm); mobile phase, acetonitrile-water (45:55, v/v); column temperature, 40 ℃; flow rate, 1.0 mL/min; detector, diode array detector (DAD); detection wavelength, 246 nm; injection volume, 10 μL. The external standard method was used to quantify the budesonide enantiomers. Under the optimized conditions, the enantiomers were well separated, and the retention times of 22*R*-budesonide and 22*S*-budesonide were 6.40 min and 7.77 min, respectively. The resolution of the enantiomers was 4.64. The linear ranges of 22*R*-budesonide and 22*S*-budesonide were 0.16-1000 μg/mL and 0.20-1000 μg/mL, respectively. The peak area of the enantiomers showed a good linear relationship with the corresponding concentration, and the correlation coefficients (*R*^2^) were 0.9999. The limits of detection (LODs) of 22*R*-budesonide and 22*S*-budesonide were 0.05 μg/mL and 0.07 μg/mL, respectively, based on a signal-to-noise ratio of 3. The limits of quantification (LOQs) were calculated to be 0.16 μg/mL and 0.20 μg/mL, respectively, based on a signal-to-noise ratio of 10. The recoveries at four spiked levels were in the range of 102.63% to 104.17%, with the relative standard deviations (RSDs) of 0.08% to 0.57% (*n*=6). The budesonide solution was stored in dark at 4 ℃ for 24 h, and no obvious degradation was observed. Finally, the method was applied to determine four actual samples of budesonide suspension for inhalation in a batch. The samples were dissolved in methanol, filtered through a 0.45 μm microporous membrane, and then analyzed. The amounts of 22*R*-budesonide and 22*S*-budesonide in the samples were in the ranges of 283.15-284.63 μg/mL and 259.86-261.51 μg/mL, respectively. This method is simple and rapid, in addition to having good repeatability and high accuracy. It can be used for the resolution of budesonide enantiomers and for quality control in budesonide preparations.

布地奈德(budesonide)是一种具有高效局部抗炎作用的糖皮质激素,因其显著的首过效应,多以呼吸道给药的方式用于治疗哮喘和溃疡性结肠炎^[1,2,3,4]^。药物中布地奈德主要以22*R*和22*S*异构体的形式存在(结构式见图1),其质量比约为1:1,且22*R*的药物活性比22*S*强2~3倍^[5]^。因此,从药物研发及质量控制角度看,开发布地奈德对映体拆分和定量分析方法具有重要意义。

**图1:**
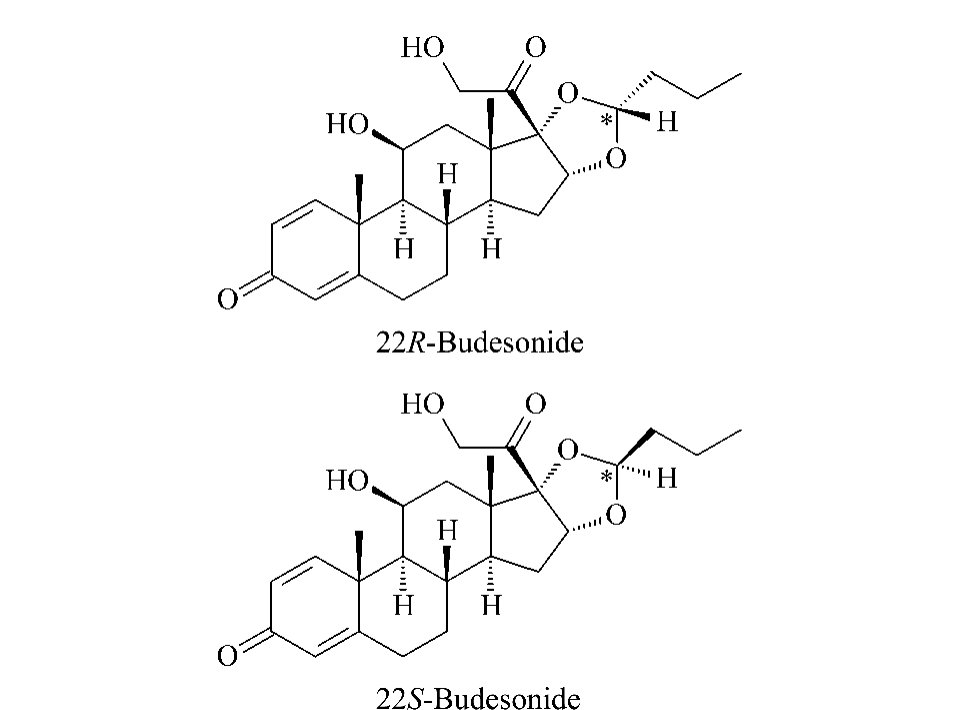
布地奈德对映体的化学结构式

目前,文献报道的布地奈德定量分析方法主要有高效液相色谱法和液相色谱-串联质谱法,其固定相主要是反相C_18_柱。如周清等^[6]^和Demurtas等^[7]^均以反相C_18_柱为固定相,甲醇-水为流动相,建立了布地奈德高效液相色谱定量分析方法,并对实际样品进行了测定,但均未对布地奈德对映体进行拆分;Alkhateeb等^[8]^以反相C_18_柱为固定相,乙腈-20 mmol/L乙酸铵(含0.1%氢氧化铵)为流动相,建立了高效液相色谱法,实现了对布地奈德对映体及其相关杂质的拆分,其色谱图表明布地奈德对映体可实现基线分离;Lu等^[9]^和Li等^[10]^均以反相C_18_柱为固定相,分别以乙腈-5 mmol/L乙酸铵(含0.14%乙酸)和乙醇-水为流动相,建立了布地奈德对映体的液相色谱-串联质谱定量分析方法,并对血浆中的布地奈德对映体进行了测定,其总离子流色谱图显示布地奈德对映体可实现基线分离。

采用手性固定相拆分手性化合物已有较多报道^[11,12,13,14,15]^,但对布地奈德对映体的拆分却少有报道。本文基于直链淀粉-三[(*S*)-1-苯乙基氨基甲酸酯]手性固定相,建立了布地奈德对映体高效液相色谱定量分析方法,考察了流动相组成和柱温对对映体保留时间、色谱峰高、色谱峰宽和分离度的影响,并对吸入用布地奈德混悬液实际样品进行测定。

## 1 实验部分

### 1.1 仪器、试剂与材料

Agilent 1260高效液相色谱仪(配有DAD检测器)和0.45 μm微孔滤膜(美国Agilent公司); XP105天平(上海Mettler-Toledo公司)。

乙腈和甲醇(HPLC级,德国Merck KGaA公司);高氯酸、甲酸、乙酸、乙酸铵、22*R*-布地奈德和22*R*,*S*-布地奈德对照品(分析纯,上海Macklin Biochemical公司);市售吸入用布地奈德混悬液1批次;实验用水为超纯水。

### 1.2 标准溶液的配制

标准储备溶液:分别准确称取适量22*R*-布地奈德和22*R*,*S*-布地奈德对照品,用甲醇溶解并定容,分别配制成1000 μg/mL和2000 μg/mL的标准储备液,于4 ℃避光保存,现用现配。

标准工作溶液:用甲醇将22*R*-布地奈德和22*R*,*S*-布地奈德标准储备溶液逐级稀释成适当浓度的标准工作溶液,于4 ℃避光保存。

### 1.3 供试品溶液的配制

取吸入用布地奈德混悬液一瓶,充分摇匀后开瓶,将瓶内混悬液移入10 mL容量瓶中,用甲醇润洗瓶内壁3次,并将洗液移入容量瓶中,最后用甲醇定容,配制成500 μg/mL供试品溶液,于4 ℃避光保存。检测时,供试品溶液过0.45 μm微孔滤膜后进样分析。

### 1.4 液相色谱条件

色谱柱:Chiralpak AS-RH色谱柱(150 mm×4.6 mm, 5.0 μm,日本Daicel公司);柱温:40 ℃;流动相:乙腈-水(45:55, v/v);流速:1.0 mL/min;检测波长:246 nm;进样量:10 μL。

## 2 结果与讨论

### 2.1 色谱条件的选择

2.1.1 色谱柱的选择

Chiralpak AS-RH色谱柱和Chiralpak AD-RH色谱柱(150 mm×4.6 mm, 5.0 μm,日本Daicel公司)的手性固定相分别为直链淀粉-三[(*S*)-1-苯乙基氨基甲酸酯]和直链淀粉-三(3,5-二甲基苯基氨基甲酸酯),本文考察了这两种色谱柱对布地奈德对映体的拆分效果,色谱图见图2。实验中保持22*R*-布地奈德和22*R*,*S*-布地奈德标准工作溶液浓度、流速、进样量和检测波长等参数一致,流动相为乙腈-水(45:55, v/v),柱温为25 ℃。结果表明,采用两种手性固定相色谱柱时,22*R*-布地奈德均先于22*S*-布地奈德出峰,与文献^[9,16,17]^报道采用反相C_18_固定相的出峰顺序一致;该流动相下布地奈德对映体在Chiralpak AS-RH色谱柱上的分离度、色谱峰形、色谱峰高等均优于Chiralpak AD-RH色谱柱;采用Chiralpak AS-RH色谱柱时布地奈德对映体在10 min内可实现完全分离。因此最终选择Chiralpak AS-RH色谱柱为实验用色谱柱。

**图2:**
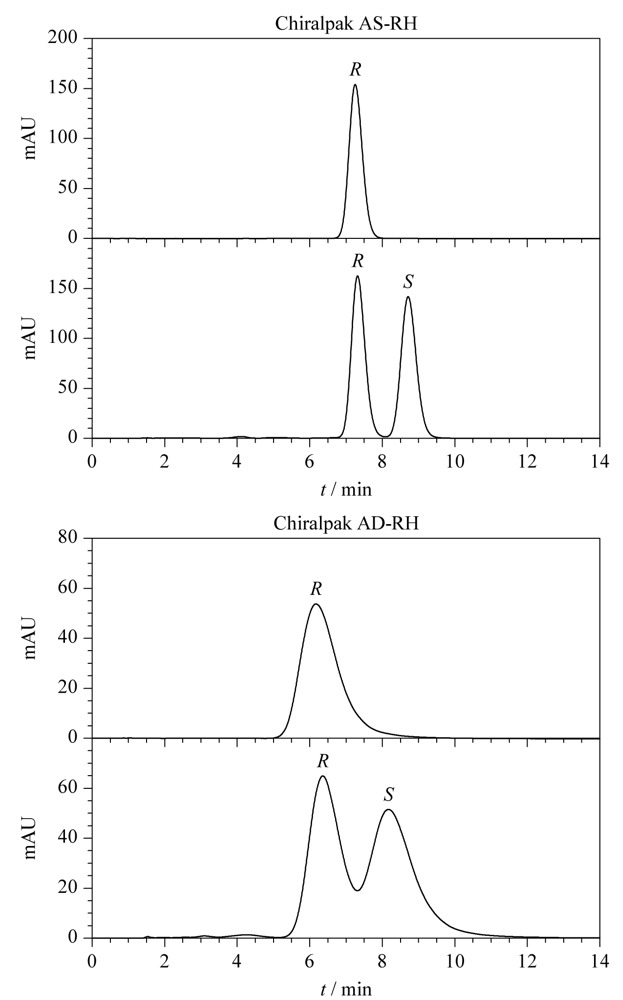
不同色谱柱上布地奈德对映体的色谱图

2.1.2 流动相对布地奈德拆分的影响

一般调整流动相体系的pH值,可以建立稳定的溶质-固定相作用环境,从而优化目标物保留时间和色谱峰形^[18]^。通过分析文献^[7,9,19-21]^报道的反相C_18_固定相用于分析布地奈德的流动相组成,本文重点考察了甲醇-水(45:55, v/v)、乙腈-5 mmol/L乙酸铵(含0.14%乙酸)(45:55, v/v)、乙腈-0.10%甲酸水溶液(45:55, v/v)、乙腈-水(45:55, v/v)和乙腈-0.25%高氯酸(45:55, v/v)对布地奈德对映体的拆分效果,色谱图见图3。实验中保持22*R*,*S*-布地奈德标准工作溶液浓度、流速、进样量和检测波长等参数一致,固定相为Chiralpak AS-RH色谱柱,柱温为25 ℃。结果表明,在乙腈-水流动相体系中添加甲酸、乙酸和乙酸铵时,布地奈德对映体的色谱峰保留时间、色谱峰高、色谱峰宽和分离度均无明显影响,添加高氯酸时,布地奈德对映体的保留时间均变小,但色谱峰高、色谱峰宽和分离度也无明显改善,这说明常用的流动相添加剂对布地奈德对映体的拆分无明显的改善效果;而采用甲醇-水体系时,20 min内无法洗脱布地奈德对映体,其原因还有待进一步研究。因此最终选择乙腈-水(45:55, v/v)为实验用流动相。

**图3:**
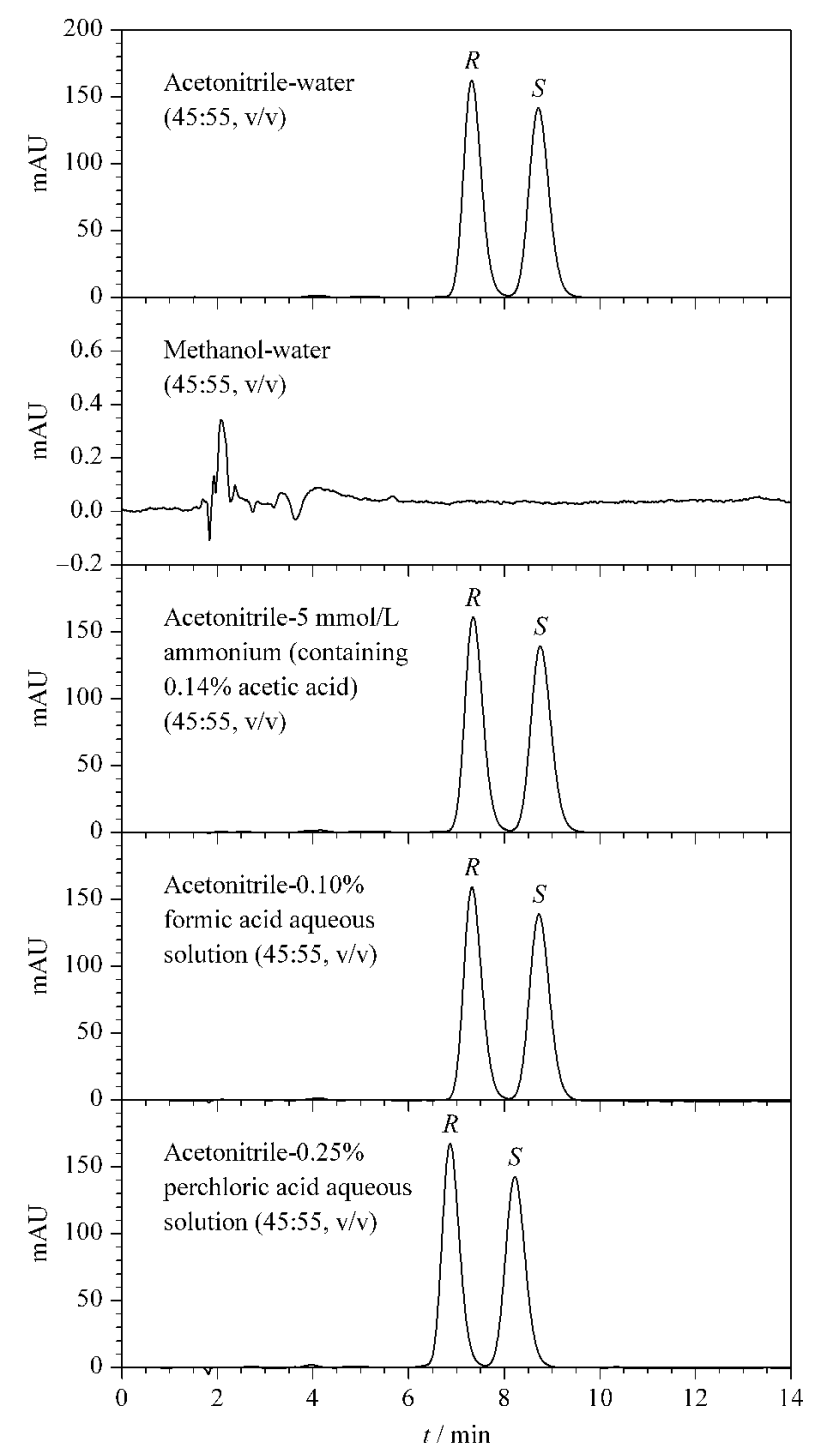
不同流动相下布地奈德对映体的色谱图

2.1.3 柱温对布地奈德拆分的影响

本文以Chiralpak AS-RH色谱柱为固定相,乙腈-水(45:55, v/v)为流动相,保持22*R*,*S*-布地奈德标准工作溶液浓度、流速、进样量和检测波长等参数一致的条件下,考察了柱温(25~40 ℃)对布地奈德对映体拆分的影响,色谱图见图4。结果显示,随着柱温的升高,布地奈德对映体的保留时间和色谱峰宽呈下降趋势,色谱峰高和分离度呈上升趋势,升高柱温可以明显改善布地奈德对映体的拆分效果;当柱温为40 ℃时,22*R*-布地奈德和22*S*-布地奈德的保留时间分别6.40 min和7.77 min,分离度为4.64。因此最终选择40 ℃为实验用柱温。

**图4:**
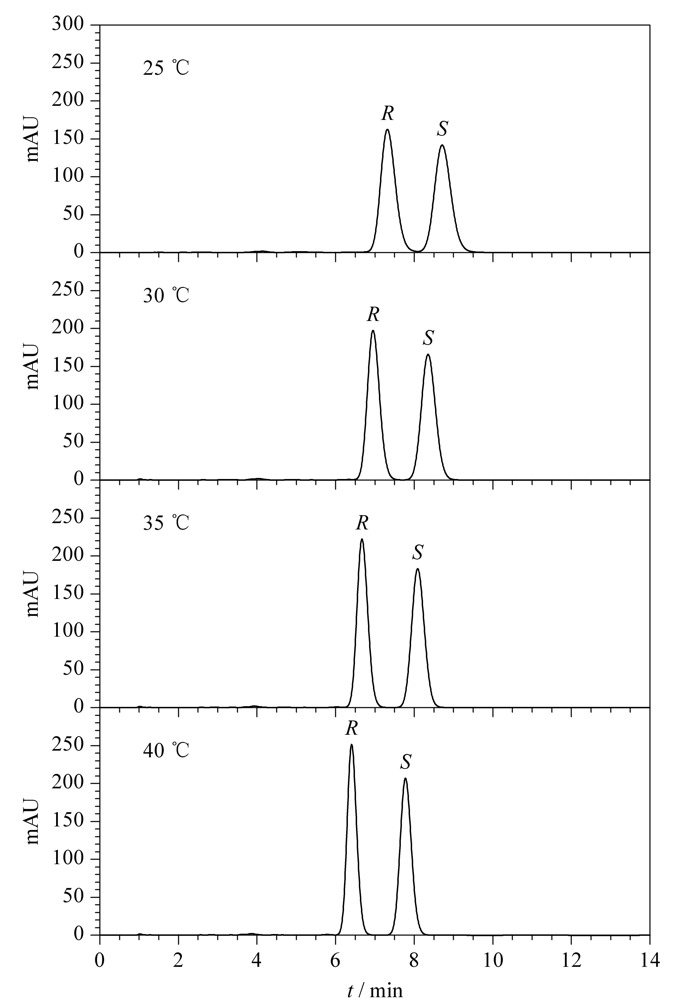
不同柱温下布地奈德对映体的色谱图

化合物对映体间的分离度随柱温升高增大或减小均有相关文献^[22,23,24,25,26,27,28,29]^报道。液相色谱手性拆分中,对映体之间分离因子*α*可用如下公式^[24,30]^表示:

ln *α*=- $\frac{\Delta \Delta H^{0}}{RT}$ + $\frac{\Delta \Delta S^{0}}{R}$

其中,R为气体常数(8.314 J/(mol·K)); *T*为柱温(K); ΔΔ*H*^0^和ΔΔ*S*^0^分别为对映体在固定相和流动相间分配的焓变之差(J/mol)和熵变之差(J/(mol·K))。可以看出,对映体的分离主要由焓变和熵变共同控制,若柱温升高分离度变好,一般为熵变控制,反之则为焓变控制^[24]^。因此,对映体的分离,不仅与对映体分子结构有关,也与固定相和流动相种类有关。

### 2.2 方法的线性范围、检出限和定量限

采用1.4节的液相色谱条件对22*R*-布地奈德标准工作溶液进行测定,以分析物的峰面积(*Y*)和对应的质量浓度(*X*, μg/mL)进行线性回归,得到22*R*-布地奈德标准曲线和相关系数(*R*^2^),以色谱峰*S/N*≥3和10时目标物的水平为方法的检出限和定量限。然后,以22*R*-布地奈德为对照品,测定22*R*,*S*-布地奈德对照品中22*R*-布地奈德的含量为51%,即22*R*,*S*-布地奈德对照品中22*S*-布地奈德的含量为49%;最后,再采用1.4节的液相色谱条件对22*R*,*S*-布地奈德标准工作溶液进行测定,得到22*S*-布地奈德的标准曲线、相关系数、检出限和定量限,结果见表1。结果表明,22*R*-布地奈德和22*S*-布地奈德在对应的范围内线性关系良好,相关系数均为0.9999,检出限分别为0.05 μg/mL和0.07 μg/mL,定量限分别为0.16 μg/mL和0.20 μg/mL。

**表1:** 布地奈德对映体的线性范围、线性方程、相关系数、检出限和定量限

Budesonide enantiomer	Linear range/(μg/mL)	Linear equation	R^2^	LOD/(μg/mL)	LOQ/(μg/mL)
R	0.16-1000	Y=18.51X+7.68	0.9999	0.05	0.16
S	0.20-1000	Y=18.72X+8.66	0.9999	0.07	0.20

*Y*: peak area; *X*: mass concentration, μg/mL.

### 2.3 加标回收率和精密度

为了验证该方法的准确度和精密度,综合考虑标准曲线的线性范围和供试品溶液中布地奈德对映体的含量,实验向供试品溶液中添加4个水平的22*R*,*S*-布地奈德对照品溶液,每个加标样品测量6次,计算加标回收率和精密度,具体结果见表2。结果表明,平均回收率为102.63%~104.17%,相对标准偏差为0.08%~0.57%。该方法准确可靠,能够满足实际分析的要求。

**表2:** 布地奈德对映体的加标回收率及相对标准偏差(*n*=6)

Budesonide enantiomer	Background/ (μg/mL)	Added/ (μg/mL)	Found/ (μg/mL)	Recovery/ %	RSD/ %
R	284.63	192.35	482.63	102.94	0.13
		216.40	506.73	102.63	0.57
		240.45	531.53	102.68	0.48
		360.70	655.58	102.84	0.12
S	261.51	180.45	447.26	102.94	0.18
		203.00	470.46	102.93	0.55
		225.55	494.46	103.28	0.51
		338.30	613.91	104.17	0.08

### 2.4 稳定性

采用1.4节液相色谱条件,分别对于4 ℃避光保存0、4、8、12和24 h的布地奈德溶液进样测定,22*R*-布地奈德和22*S*-布地奈德峰面积的RSD分别为0.27%和0.31%,表明布地奈德溶液在本实验条件下24 h内稳定。

### 2.5 实际样品的测定

采用本方法对1批次共4个吸入用布地奈德混悬液样品进行测试,每个样品均采用1.3节的方法进行配制,样品色谱图见图5。结果表明,布地奈德混悬液的辅料对其对映体的测定不产生明显干扰,供试品中22*R*-布地奈德、22*S*-布地奈德及其总含量分别为283.15~284.63 μg/mL、259.86~261.51 μg/mL和543.01~546.14 μg/mL, RSD为0.26%(*n*=3)。

**图5:**
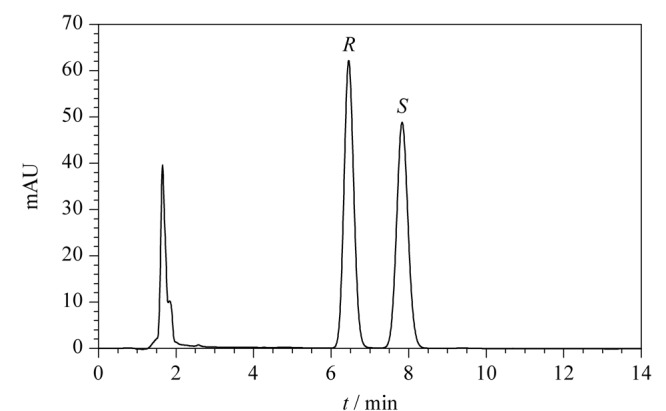
实际样品的色谱图

## 3 结论

本文以直链淀粉-三[(*S*)-1-苯乙基氨基甲酸酯]为手性固定相,实现了布地奈德对映体的快速拆分,建立了一种测定布地奈德对映体含量的高效液相色谱方法,并应用于吸入用布地奈德混悬液实际样品的检测。该方法具有流动相简单、拆分效果好、分析时间短、线性范围宽等优点,为布地奈德对映体的快速拆分和其制剂的质量监测提供了简单、实用的方法。
